# Basophils from Cancer Patients Respond to Immune Stimuli and Predict Clinical Outcome

**DOI:** 10.3390/cells9071631

**Published:** 2020-07-07

**Authors:** Heather J. Bax, Jitesh Chauhan, Chara Stavraka, Atousa Khiabany, Mano Nakamura, Giulia Pellizzari, Kristina M. Ilieva, Sara Lombardi, Hannah J. Gould, Christopher J. Corrigan, Stephen J. Till, Sidath Katugampola, Paul S. Jones, Claire Barton, Anna Winship, Sharmistha Ghosh, Ana Montes, Debra H. Josephs, James F. Spicer, Sophia N. Karagiannis

**Affiliations:** 1 St. John’s Institute of Dermatology, School of Basic & Medical Biosciences, King’s College London, London SE1 9RT, UK; heather.bax@kcl.ac.uk (H.J.B.); jitesh.chauhan@kcl.ac.uk (J.C.); chara.stavraka@gstt.nhs.uk (C.S.); atousa.khiabany@outlook.com (A.K.); mano.nakamura@kcl.ac.uk (M.N.); giulia.pellizzari@kcl.ac.uk (G.P.); kristina.ilieva@kcl.ac.uk (K.M.I.); debra.josephs@gstt.nhs.uk (D.H.J.); 2 School of Cancer & Pharmaceutical Sciences, King’s College London, Guy’s Hospital, London SE1 9RT, UK; james.spicer@kcl.ac.uk; 3 Departments of Medical Oncology and Clinical Oncology, Guy’s and St Thomas’ NHS Foundation Trust, London SE1 9RT, UK; anna.winship@gstt.nhs.uk (A.W.); sharmistha.ghosh@gstt.nhs.uk (S.G.); ana.montes@gstt.nhs.uk (A.M.); 4 Breast Cancer Now Research Unit, School of Cancer & Pharmaceutical Sciences, King’s College London, Guy’s Cancer Centre, London SE1 9RT, UK; 5 Guy’s and St Thomas’ Oncology & Haematology Clinical Trials (OHCT), Guy’s Cancer Centre, London SE1 9RT, UK; sara.lombardi@kcl.ac.uk; 6 Randall Centre for Cell and Molecular Biophysics, School of Basic and Medical Biosciences, King’s College London, London SE1 9RT, UK; hannah.gould@kcl.ac.uk; 7 Asthma UK Centre, Allergic Mechanisms in Asthma, King’s College London, London SE1 9RT, UK; chris.corrigan@kcl.ac.uk (C.J.C.); stephen.till@kcl.ac.uk (S.J.T.); 8 Department of Respiratory Medicine and Allergy and School of Immunology and Microbial Sciences, King’s College London, London SE1 9RT, UK; 9 Centre for Drug Development, Cancer Research UK, 2 Redman Place, London E20 1JQ, UK; sidathkatu@hotmail.com (S.K.); paul.jones2@ucb.com (P.S.J.); claire@barton-oncology.com (C.B.); 10 Barton Oncology Ltd., 8 Elm Avenue, Eastcote, Middlesex HA4 8PD, UK

**Keywords:** basophils, BAT, ovarian cancer, hypersensitivity, IgE, CD63, biomarkers, survival, antibodies, chemotherapy

## Abstract

Basophils are involved in manifestations of hypersensitivity, however, the current understanding of their propensity for activation and their prognostic value in cancer patients remains unclear. As in healthy and atopic individuals, basophil populations were identified in blood from ovarian cancer patients (*n* = 53) with diverse tumor histologies and treatment histories. Ex vivo basophil activation was measured by CD63 expression using the basophil activation test (BAT). Irrespective of prior treatment, basophils could be activated by stimulation with IgE- (anti-FcεRI and anti-IgE) and non-IgE (fMLP) mediated triggers. Basophil activation was detected by ex vivo exposure to paclitaxel, but not to other anti-cancer therapies, in agreement with a clinical history of systemic hypersensitivity reactions to paclitaxel. Protein and gene expression analyses support the presence of basophils (CCR3, CD123, FcεRI) and activated basophils (CD63, CD203c, tryptase) in ovarian tumors. Greater numbers of circulating basophils, cells with greater capacity for ex vivo stimulation (*n* = 35), and gene signatures indicating the presence of activated basophils in tumors (*n* = 439) were each associated with improved survival in ovarian cancer. Circulating basophils in cancer patients respond to IgE- and non-IgE-mediated signals and could help identify hypersensitivity to therapeutic agents. Activated circulating and tumor-infiltrating basophils may be potential biomarkers in oncology.

## 1. Introduction

Despite their small numbers in circulating white blood cells, basophils can elicit powerful effector functions, playing a key role in allergies. Several reports suggest that basophils can interact with the tumor microenvironment [1,2,3,4], however, to date, the participation of basophils in cancer immune surveillance remains insufficiently evaluated. Basophils in the circulation may also be activated to promote type I hypersensitivity following administration of anti-cancer therapies, including chemotherapies [5,6] or monoclonal antibodies, such as cetuximab [7]. Nevertheless, circulating basophil populations, their propensity for activation and degranulation, and their prognostic value have yet to be comprehensively explored in cancer patients.

The basophil activation test (BAT) [8,9,10] is widely used to study and predict type 1 hypersensitivity reactions to food [11,12,13,14,15], venom [16,17,18] and drugs [19,20,21,22,23,24,25] in the allergy field. To date, however, its application to study basophils ex vivo in the context of cancer has been limited to case reports or small studies focused on the detection of allergic reactions to chemotherapeutic agents [5,6,26,27,28,29]. Here, we conduct the first evaluation of basophils in a cohort of 53 ovarian cancer patients with diverse tumor histologies and treatment histories (treatment-naïve, chemotherapy, debulking surgery, targeted monoclonal antibodies (mAbs), small molecule inhibitors). We examined whether basophils from cancer patients could be identified in whole blood samples, whether basophils could be activated by IgE- and non-IgE-mediated mechanisms, and if patients’ prior treatment history could affect basophil activation. We confirmed the utility of the BAT in detecting hypersensitivity to therapeutic agents, such as the chemotherapy paclitaxel. Furthermore, we explored the prognostic value of circulating basophils in our cohort, the presence of basophils and their activation markers in tumors and the prognostic value of tumor-infiltrating basophils and their activation status, in relation to clinical outcomes.

## 2. Materials and Methods

### 2.1. Ovarian Cancer Patient Study

This study has been reviewed and approved by the Guy’s Research Ethics Committee (Reference 09/H0804/45). Ovarian cancer patients were enrolled by written, informed consent. Peripheral venous blood was collected in BD Vacutainer™ Hemogard Closure Plastic K2-EDTA Tubes (BD, Wokingham, UK). Serum samples were prepared by centrifugation of clotted blood in SST Clot Activator and Polymer Gel Hemogard Closure Blood Tubes (BD, Wokingham, UK) at 2500RPM for 15 min at 4 °C and stored at −80 °C until analysis. Serum tryptase (ng/mL) and total IgE concentrations (kU/L) were evaluated by Viapath Analytics (London, UK). Patient demographics, tumor histology, prior treatment history, and patient survival data were collected from clinical databases and anonymized. Prior treatments comprised of standard care or, in the case of the anti-PD-L1 mAb, avelumab, through the JAVELIN OVARIAN 100 trial (ClinicalTrials.gov Identifier NCT02718417).

### 2.2. Basophil Phenotyping

FcεRI expression and endogenous FcεRI-bound IgE on basophils were evaluated by incubation of unfractionated whole blood with unconjugated anti-human FcεRI and anti-human IgE mouse IgG (clone AER-37, diluted 1:50, and clone MHE-18, diluted 1:10, respectively, Biolegend, San Diego, CA, USA) (45 min, 4 °C), followed by anti-mouse IgG-FITC secondary (diluted 1:50, QIFIKIT^®^, Agilent Dako, Santa Clara, CA, USA) (30 min, 4 °C), and then, anti-CCR3-APC (clone 5E8, diluted 1:10, Biolegend, San Diego, CA, USA) (10 min, 4 °C). Expression of FcεRI and the level of endogenous receptor-bound IgE were evaluated in the CCR3-APC^high^SSC^low^ gated basophil population in unfractionated whole blood. The numbers of FcεRI and IgE molecules (per basophil) were quantified using the QIFIKIT^®^ bead cocktail plus anti-mouse IgG-FITC secondary (QIFIKIT^®^, Agilent Dako, Santa Clara, CA, USA).

### 2.3. Basophil Activation Test (BAT)

Basophil Activation Tests (BAT, Flow2 CAST^®^ kit, Bühlmann Laboratories AG, Schönenbuch, Switzerland) were performed, according to the manufacturer’s instructions, within 4 h of blood collection, unless otherwise stated. Unfractionated whole blood was incubated with stimulation buffer (Bühlmann Laboratories AG, Schönenbuch, Switzerland) and anti-FcεRI (Bühlmann Laboratories AG, Schönenbuch, Switzerland), anti-IgE antibody (Agilent Dako, Santa Clara, CA, USA) or fMLP (Bühlmann Laboratories AG, Schönenbuch, Switzerland). Ex vivo stimulation with anti-cancer therapies—paclitaxel (Pfizer, Sandwich, UK), carboplatin (Hospira, Maidenhead, UK), cetuximab (Erbitux^®^, Merck, Darmstadt, Germany)—was performed at concentrations ranging from 1.5 to 500 μg/mL. Samples were stained with anti-CCR3-PE and anti-CD63-FITC staining cocktail (Bühlmann Laboratories AG, Schönenbuch, Switzerland) and incubated at 37 °C for 30 min in a 5% CO_2_ incubator (incubation time was optimized from the suggested 10 min by the Flow2 CAST^®^ kit manufacturers (Bühlmann Laboratories AG, Schönenbuch, Switzerland), with the maximal activation achieved after 30 min incubation). Following red blood cell lysis (Bühlmann Laboratories AG, Schönenbuch, Switzerland) (10 min, room temperature), samples were centrifuged, and cell pellets were resuspended with acquisition buffer (Bühlmann Laboratories AG, Schönenbuch, Switzerland). Basophil populations were determined as % gated CCR3-PE^high^SSC^low^ basophils in total white blood cells (WBC) in unfractionated whole blood. Basophil activation was expressed as Stimulation Index (SI; fold change in % CD63-positive CCR3-PE^high^SSC^low^ basophils over background control (stimulation buffer and staining antibody cocktail alone) for each sample).

We investigated whether the capacity for ex vivo activation of basophils in unfractionated whole blood was influenced by stimulation 0–4 (*n* = 8), 24 (*n* = 8) or 48 h (*n* = 5) after sample collection. Similar proportions of basophils in whole blood were identified in matched blood samples at all time points, and similarly to basophil activation by anti-IgE in blood from healthy, non-atopic and atopic subjects [10,30,31], cancer patient basophils stored for up to 48 h following blood collection could be activated ex vivo by IgE- and non-IgE-dependent mechanisms, albeit with some attenuation of the response to IgE-dependent activation (Supplementary Materials, Figure S1).

### 2.4. Flow Cytometric and Statistical Analyses

All flow cytometric acquisitions were performed with a FACSCanto™ II using FACSDiva software version 8 (BD, San Diego, CA, USA). Analyses and representative plots were conducted using FlowJo™ software version 10 (FlowJo LLC, BD, San Diego, CA, USA). Statistical analyses (t-test, one-way ANOVA with Kruskal-Wallis multiple comparisons, linear regression) were performed in GraphPad Prism version 8 (GraphPad Software, Inc., San Diego, CA, USA). *p* values: * = *p* < 0.05, ** = *p* < 0.01, *** = *p* < 0.001, **** = *p* < 0.0001. Error bars represent the Standard Error of the Mean (SEM).

### 2.5. Basophil Marker Expression in Ovarian Cancer Tumors

Protein expression of basophil markers (CCR3, CD123 and FcεRI) and markers of basophil activation (CD63, CD203c, and tryptase) were studied in ovarian cancer tumors using immunohistochemistry (IHC) data available in The Pathology Atlas of The Human Protein Atlas online tool [32,33] (https://www.proteinatlas.org/humanproteome/pathology, Stockholm and Uppsala, Sweden). The antibodies used for IHC analyses are listed in Table S1. Gene expression of the same markers were studied in normal ovary and ovarian cancer tissues using the Gene Expression Profiling Interactive Analysis (GEPIA) online tool [34] (http://gepia.cancer-pku.cn/index.html, Beijing, China).

### 2.6. Survival Analyses

In our cohort of 53 ovarian cancer patients, Kaplan–Meier (KM) analyses were performed to study patient survival in association with percentage of circulating basophils (CCR3^high^SSC^low^ gated basophil population in unfractionated whole blood), the capacity of circulating basophils for ex vivo activation (stimulation index following immune stimulation), or serum tryptase concentration. Similar survival analyses were performed in association with gene expression of basophil markers in ovarian cancer patient tumors using the Kaplan–Meier (KM) Plotter online tool [35] (http://kmplot.com/analysis/index.php?p=service&cancer=ovar, Budapest, Hungary). Gene expression analyses of tumor-resident basophils (by CD123, CCR3 and FcεRI) and activated basophil signatures (by combinations of CD123, CCR3, FcεRI, CD63, CD203c and tryptase) were performed. Probes used for gene expression and datasets included in the analyses are listed in Table S2. Patients were grouped into the top tertile (T3) and lower tertile (T1), which resulted in exclusion of patients in the middle tertile (T2) and a variation in the number of patients in each group, dependent on the characteristic studied.

## 3. Results

### 3.1. Basophils Are Detectable in the Blood of Cancer Patients

**Figure 1:**
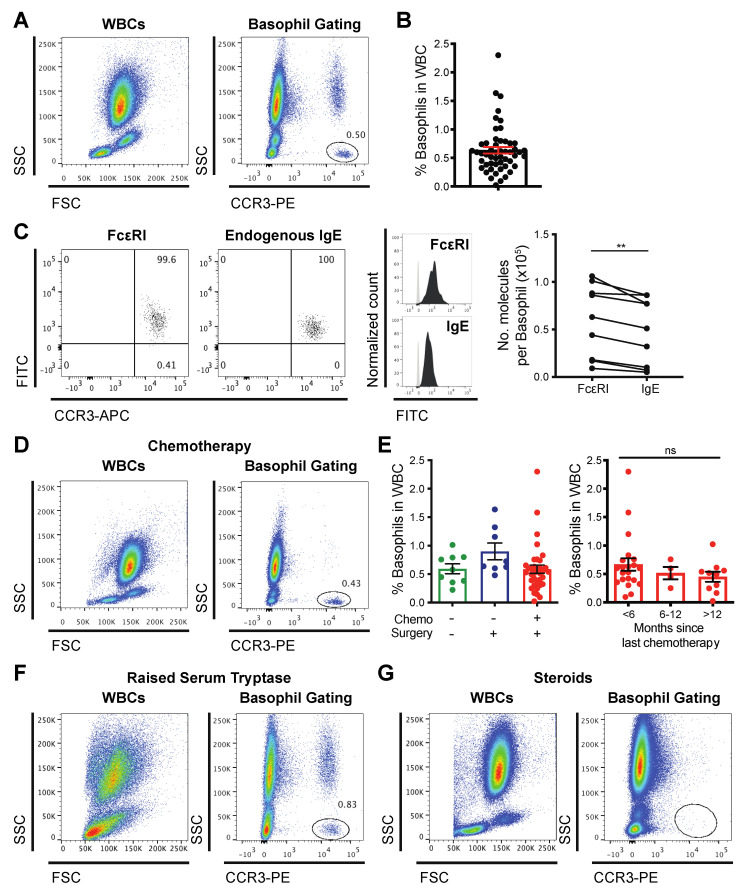
Basophils in ovarian cancer patient blood. CCR3^high^SSC^low^ basophils identified in unfractionated whole blood by flow cytometry (**A**,**B**), express FcεRI, some of which carry endogenous receptor-bound IgE antibodies (**C**). Basophil populations (gated in circle) were not impacted by prior chemotherapy (**D**), prior treatment history or time since last chemotherapy (−/+ treatment previously received or not); (**E**) or elevated serum tryptase (**F**). Recent prolonged high-dose oral corticosteroids were associated with a marked depletion of basophil populations (gated in circle) (**G**). ns = not significant; ** = *p* ≤ 0.01.

To study circulating basophils, we detected cell-surface CCR3, as this marker is expressed highly and stably, independent of the atopic status of the individual or activation state of the basophils [36]. We identified basophils (CCR3-PE^high^SSC^low^, 0.64% ± 0.06 of white blood cells (WBC), range 0.02–2.3%) in unfractionated whole blood samples from 52 of a cohort of 53 patients with ovarian cancer (Figure 1A,B). CCR3-expressing circulating basophils from patients expressed the high affinity IgE receptor, FcεRI, and carried endogenous IgE on the cell surface. We quantified the number of FcεRI and endogenous IgE molecules per basophil (by QIFIKIT^®^). The significantly higher number of FcεRI molecules per basophil, compared to endogenous IgE molecules per cell in the same blood samples, demonstrated that some FcεRI were unoccupied (Figure 1C, *n* = 9). Although chemotherapy is known to impact immune cell counts, pre-treated patients showed a clearly defined CCR3^high^SSC^low^ circulating basophil population (representative plot, Figure 1D); the proportions of basophils in white blood cells were comparable in blood from treatment-naïve patients (*n* = 9) and those who had previously undergone primary debulking surgery (*n* = 8), and prior treatment with surgery plus chemotherapy (*n* = 35). Basophil counts were independent of the time lapse since chemotherapy infusion (Figure 1E and Figure S2). CCR3^high^SSC^low^ basophils were clearly identifiable in a blood sample from a patient with elevated serum tryptase (Figure 1F), but not from a patient who had received a recent prolonged course of high-dose oral corticosteroids (Figure 1G). In summary, basophils could be clearly identified in 98% of unfractionated blood samples from a diverse cohort of ovarian cancer patients, irrespective of treatment.

### 3.2. Basophils from Cancer Patients Can Be Activated by IgE and Non-IgE-Mediated Triggers Ex Vivo

**Figure 2:**
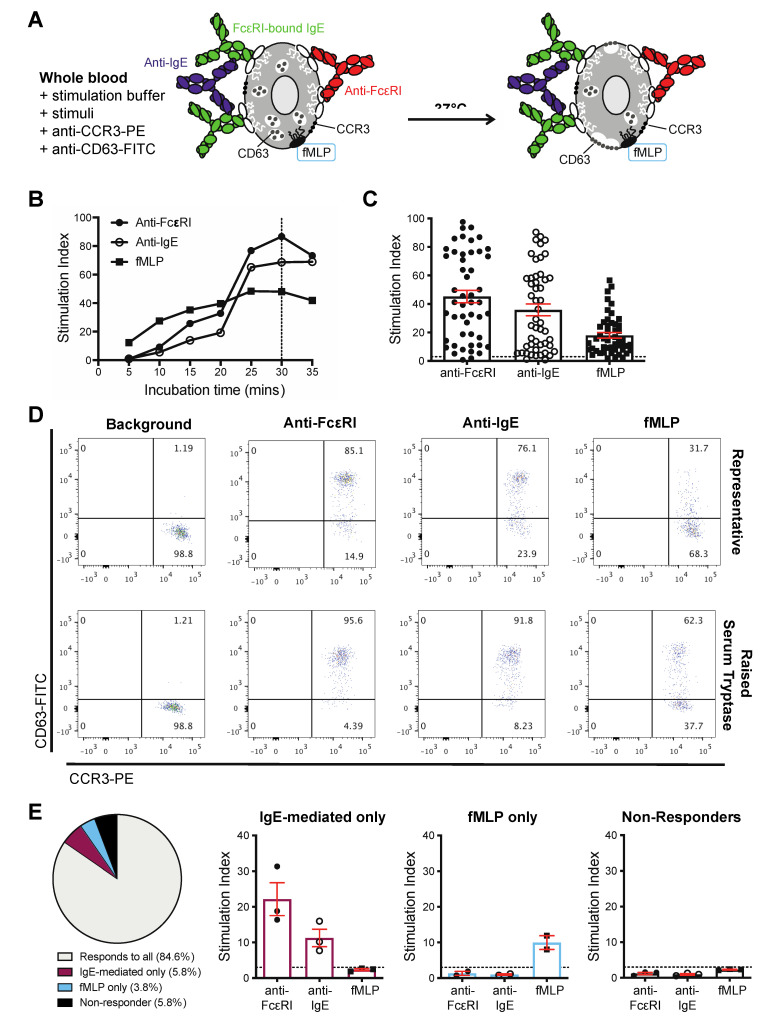
Basophil stimulation ex vivo. Cell-surface CD63 up-regulation triggered by IgE- and non-IgE-mediated stimuli (**A**). Stimulation for 5–35 min (**B**). Activation (≥3.0 Stimulation Index; cut-off: dotted line), was induced by IgE-mediated; anti-FcεRI and anti-IgE, and/or non-IgE-mediated; fMLP, stimulation (**C**,**D**). Analyses of basophil responses to none (“non-responders”), one or more stimulants (**E**).

We next investigated the capacity of basophils from ovarian cancer patients to respond to established external IgE- and non-IgE-dependent activation and degranulation stimuli. Having shown that the basophils expressed FcεRI and carried endogenous IgE, we evaluated IgE-mediated activation using polyclonal anti-FcεRI and anti-IgE antibodies, as well as non-IgE mediated activation using the bacterial-derived peptide, fMLP. We monitored up-regulation of CD63 on the surface of CCR3^high^SSC^low^ basophils (Figure 2A). CD63 is a well-established marker of basophil activation [37,38,39], and it is known to correlate with degranulation and histamine release in response to stimulation [40,41,42,43]. CD63 up-regulation increased with time of stimulation with maximal activation measured at 30 min (Figure 2B), which is in concordance with observations of activation of basophils in samples from allergic patients [13]. In the cohort of 52 evaluable samples from patients with different cancer histologies (e.g., serous, endometrioid, clear cell, carcinosarcoma, mucinous, mixed) and diverse treatment histories, circulating basophils from 49/52 (94%) of samples responded to one or more of the three stimuli (anti-FcεRI, anti-IgE, fMLP) by up-regulating cell surface CD63 (Figure 2C,D and Table S3). A high level of basophil activation was triggered by each of the three stimuli in a patient with elevated serum tryptase total serum IgE concentrations (Figure 2D and Figure S3, Table S3). In 44/52 patient samples, basophils were activated by all three stimuli. In three patient samples, basophil activation was triggered by IgE-mediated stimuli but not by fMLP. In two patient samples, we detected activation by fMLP but not by either anti-FcεRI or anti-IgE. Furthermore, basophils were not significantly activated by any stimuli in samples from two patients, defined as ‘non-responders’ (Figure 2E and Table S3).

### 3.3. Patient-Derived Basophils Can Be Activated Ex Vivo Irrespective of Prior Therapy

**Figure 3:**
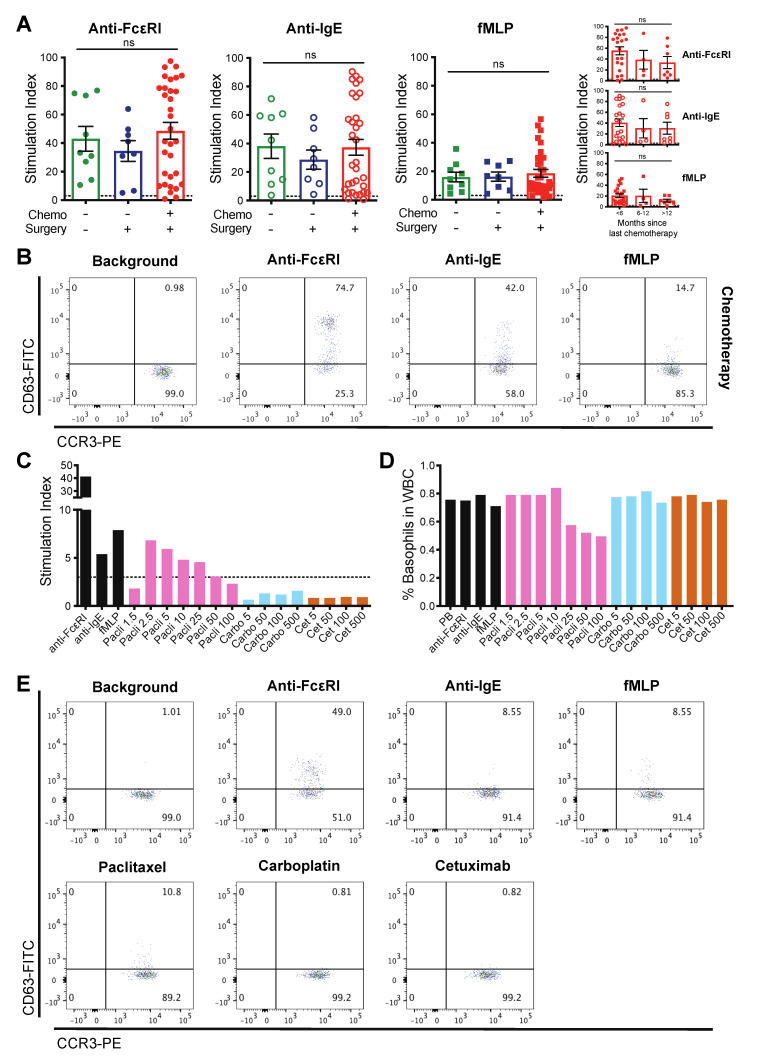
Anti-cancer treatments. Ex vivo basophil activation in blood from treatment-naïve patients, those having undergone surgery only, or surgery and chemotherapy (Stimulation Index—fold change in % CD63) (−/+ treatment previously received or not; **A**); time since last chemotherapy, right (**B**). Basophil activation by ex vivo stimulation with chemotherapy to which the patient had previously experienced systemic hypersensitivity (**C**), representative plots, (**E**), with marked basophil depletion at the highest concentrations (μg/mL; **D**). ns = not significant.

We asked whether basophils in the blood of patients with diverse treatment histories could be activated ex vivo by monitoring up-regulation of CD63 on the cell-surface. Basophil activation was triggered, by anti-FcεRI, anti-IgE, and fMLP to an equivalent degree in treatment naïve patients (*n* = 9), those who had previously undergone primary debulking surgery (*n* = 8), or surgery and chemotherapy (*n* = 32). The degree of activation was also independent of the time since the last chemotherapy treatment (Figure 3A,B and Figure S2, Table S3).

Next, we investigated whether the basophil activation test (BAT) can confirm previous hypersensitivity to a therapeutic agent in a cancer patient. In concordance with data from several case studies [5,6,26,27,28,29], clinical hypersensitivity to chemotherapy was reflected by activation following ex vivo basophil stimulation with these agents. In blood from a patient with ovarian cancer who had experienced a systemic reaction during intravenous (IV) infusion of paclitaxel over four years previously, ex vivo basophil activation was triggered following incubation with paclitaxel (2.5–25 μg/mL), to a degree comparable to that measured following stimulation with anti-IgE and fMLP (Figure 3C,E). This was despite significant treatment with a range of therapies in the intervening period. In addition, attenuation of basophil activation in the presence of the highest concentrations of paclitaxel (50–100 μg/mL), correlated with a marked reduction in basophil numbers in the unfractionated blood. We interpreted this as deleterious activation of basophils in the presence of high concentrations of this drug, which may suggest extensive degranulation, a picture reflective of previous clinical observations of hypersensitivity to this chemotherapy in this patient (Figure 3D and Figure S4). This same ovarian cancer patient was clinically tolerant to carboplatin. In concordance, basophils from this patient were not activated ex vivo by stimulation with this chemotherapy. The patient’s basophils were not activated by the anti-EGFR mAb, cetuximab, which has been widely reported to trigger hypersensitivity reactions in a subset of cancer patients who have IgE antibodies against galactose-alpha-1,3-galactose (alpha-gal), that decorates cetuximab [7,44,45,46] (Figure 3C–E).

**Figure 4:**
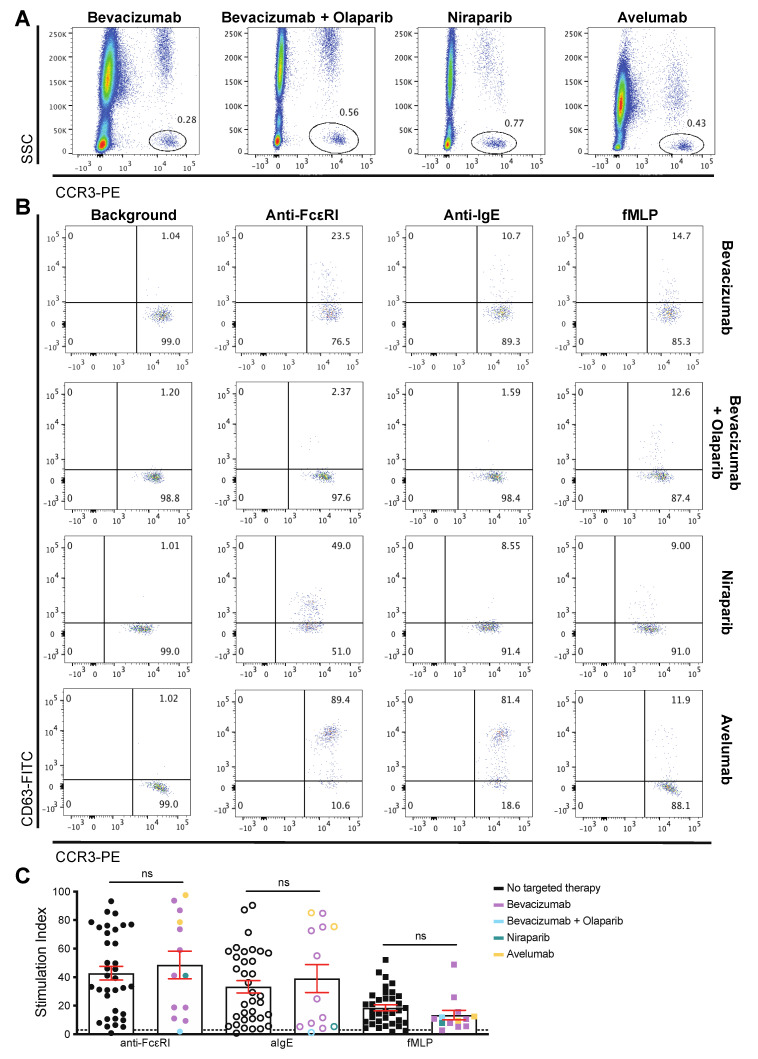
Targeted cancer therapies. Basophils were identified (gated in circles) in blood samples from ovarian cancer patients who previously received monoclonal antibodies bevacizumab (anti-VEGF) or avelumab (anti-PD-L1), or PARP inhibitors (olaparib or niraparib) (**A**). Ex vivo basophil activation levels (Stimulation Index—fold change in % CD63) triggered in the blood of these patients was comparable to patients not treated with targeted therapies (**B**,**C**). ns = not significant.

We evaluated basophils from patients who had been previously treated with targeted anti-cancer therapies: (i) the anti-VEGF mAb bevacizumab [47,48,49] (*n* = 9), the only antibody approved for the treatment of ovarian cancer, and the administration of which has been reported to trigger hypersensitivity [48,49,50]; (ii) the poly-ADP ribose polymerase (PARP) inhibitors olaparib (*n* = 1) and niraparib [51,52,53] (*n* = 1); (iii) the anti-PD-L1 mAb avelumab [47] (*n* = 2). In all patient samples, basophils were identified (Figure 4A and Figure S5A) and retained capacity to be stimulated with anti-FcεRI, anti-IgE, and fMLP to an equivalent degree to that observed in patients who had not previously received targeted therapies (Figure 4B,C and Figure S5B, Table S3).

In summary, basophils retain the capacity to be activated ex vivo irrespective of prior anti-cancer treatment, while the BAT may prove beneficial as a screen for patient hypersensitivity to cancer therapeutic agents.

### 3.4. Basophils and Their Activation Are Associated with Survival Outcomes

**Figure 5:**
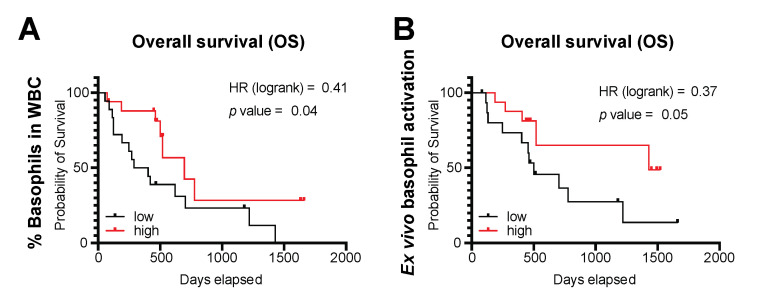
Circulating basophils and ovarian cancer patient outcomes. A higher proportion of basophils in the circulation (**A**) and a greater capacity for activation ex vivo (**B**) were associated with improved overall survival in ovarian cancer patients.

We asked whether basophils may be associated with survival outcomes in ovarian cancer. We first studied associations between patient survival and % of circulating basophils in our cohort of 53 ovarian cancer patients that we studied above in the BAT. Patients in the top tertile (T3) for the percentage of CCR3^high^SSC^low^ basophils in their blood had significantly increased overall survival compared with those in the lower tertile (T1) (Figure 5A; HR = 0.40, *p* = 0.04; median survival: T3 = 696 days (*n* = 17), T1 = 346.5 days (*n* = 18)). Overall survival was also associated with the capacity of circulating basophils to be activated ex vivo (Figure 5B; HR = 0.37, *p* = 0.05; median survival: T3 = 1429 days (*n* = 16), T1 = 501 days (*n* = 16)). 

**Figure 6:**
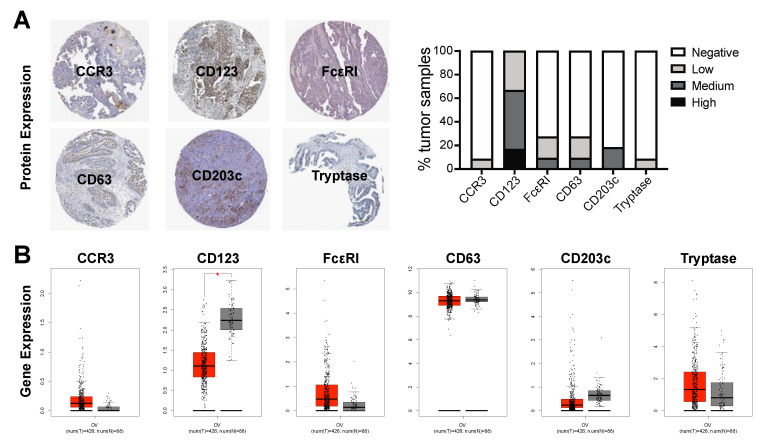
Tumor-resident basophils. Protein expression for markers of basophils (CCR3, CD123, FcεRI) and basophil activation (CD63, CD203c, tryptase) was measured in a proportion of ovarian tumors analyzed by IHC (representative images of medium staining shown; data and images from Human Protein Atlas, v19.3, proteinatlas.org) [32,33,54,55,56,57,58,59] (**A**). Similarly, gene expression for these markers was measured in both normal ovary (grey) and ovarian tumor (red) tissues [34] (**B**). * = *p* ≤ 0.05.

Next, we evaluated the presence of basophils in ovarian tumors using online tools to assess protein expression and gene expression of basophil markers. Protein expression of markers of basophils (CCR3, CD123, FcεRI) and basophil activation (CD63, CD203c, tryptase) were identified in a proportion of ovarian tumors by IHC analyses (Figure 6A) [32,33]. Furthermore, gene expression of these markers was observed in both normal ovary and ovarian tumor tissues (Figure 6B) [34]. These data suggest that basophils are found in ovarian tumors.

**Figure 7:**
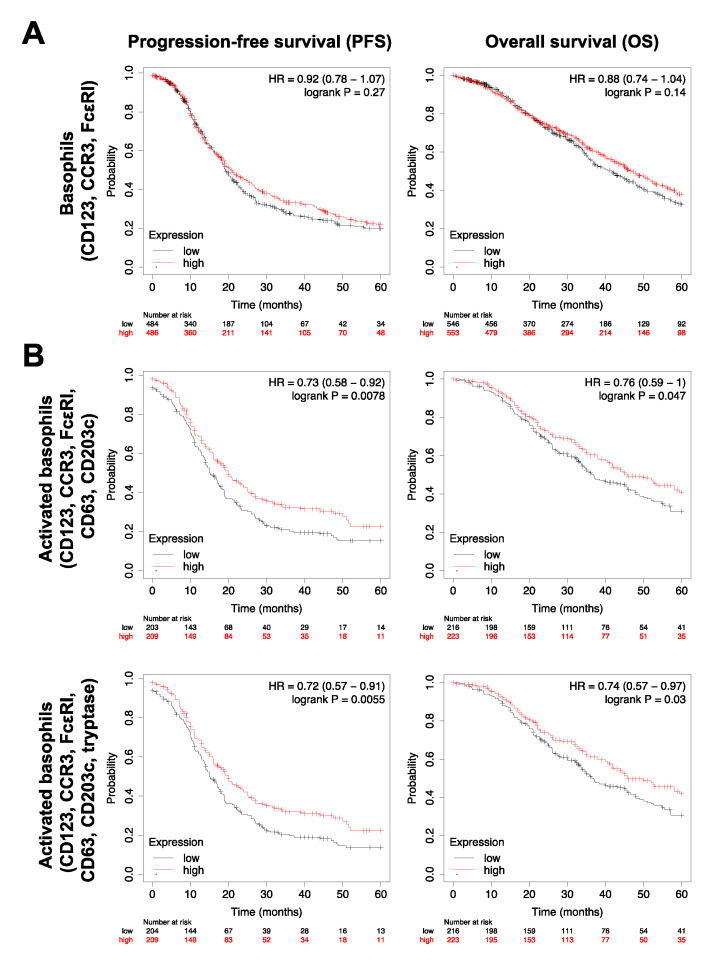
Tumor-resident basophils and ovarian cancer patient outcomes. Higher gene expression for basophils in ovarian tumors was not associated with patient survival outcomes (**A**), however, higher gene expression for activated tumor-resident basophil signatures was associated with improved progression-free and overall survival (**B**).

Having evaluated the presence of basophils in tumors, we then studied associations between patient survival outcomes and gene expression of the same basophil markers in ovarian tumors (online KM tool). This revealed that that although tumor-resident basophils (identified by CD123, CCR3 and FcεRI gene expression) were not associated with progression-free or overall survival (Figure 7A), an activated basophil signature (CD123, CCR3, FcεRI, CD63, CD203c gene expression) was significantly associated with improved outcomes (Figure 7B; PFS: HR = 0.73, *p* = 0.0078; median survival: T3 = 20 months (*n* = 209), T1 = 15.1 months (*n* = 203); OS: HR = 0.76, *p* = 0.047; median survival: T3 = 45.8 months (*n* = 223), T1 = 36.8 months (*n* = 216)). Despite there being no prognostic value of tryptase concentration alone, either in the circulation or tumor (Figure S6), improved patient prognosis was maintained when the gene signature for activated basophils in ovarian tumors included tryptase (CD123, CCR3, FcεRI, CD63, CD203c and tryptase gene expression) (Figure 7B; PFS: HR = 0.72, *p* = 0.0055; median survival: T3 = 20 months (*n* = 209), T1 = 15 months (*n* = 204); OS: HR = 0.74, *p* = 0.03; median survival T3 = 45.8 months (*n* = 223), T1 = 36.8 months (*n* = 216)).

Together, these findings suggest that activated basophils, either in the circulation or tumor, are associated with a survival benefit in ovarian cancer and that, largely independently of prior clinical treatment, blood basophils can be identified and stimulated to degranulate and used to confirm hypersensitivity to chemotherapies.

## 4. Discussion

Tumors and chemotherapeutic agents are known to affect peripheral blood immune cells. Rare populations such as circulating basophils in patients with cancer and their functional capacity for activation are insufficiently studied. Although previous evidence suggests that human basophils may be refractory to immune regulation known to affect the functions of other immune cells [40], little is known about the potential of blood basophils from patients with cancer to retain their capacity for activation. Here, we identified a discrete population of CCR3^high^SSC^low^ basophils in the blood of patients with ovarian cancer of diverse histologies and treatment histories (Table S3). Like basophils from healthy and atopic individuals [60,61,62,63,64], the basophils from ovarian cancer patients also expressed cell-surface FcεRI, which were partly occupied by endogenous IgE. Having confirmed this basophil phenotype, we showed that patient-derived basophils were susceptible to activation by IgE- and non-IgE-mediated stimuli irrespective of prior anti-cancer therapy.

In our study of circulating basophils from ovarian cancer patients, we selected CCR3 as a marker for basophil identification as it is routinely used in BAT assays and has been previously described as a stable marker, which is highly expressed, independent of the atopic status of the individual or activation state of the basophils. This allowed for accurate basophil identification without the need for a second marker [36]. Furthermore, cells identified by CCR3 expression have high concordance with those identified with marker combinations, such as CCR3+/CD3-, and CRTH2+/CD203c+/CD3- populations [65]. We monitored up-regulation of CD63 on the surface of basophils following stimulation, since this marker of basophil activation is well established [37,38,39], and is known to correlate with degranulation and histamine release in response to stimulation [40,41,42,43]. However, future studies of circulating basophils in cancer may benefit from the inclusion of additional markers for basophil identification (such as CD203c, CD123, and CRTH2), and basophil activation (such as CD203c, CD107a, CD13, CD164, CD69, and histamine or tryptase release) [9,42].

Although chemotherapy typically reduces blood immune cell counts, 98% of our 53 patients had a clearly identifiable circulating basophil population (Figure 1). This was largely independent of prior cancer therapy, except for one patient who had received recent prolonged, high-dose corticosteroids prior to sampling and whose blood was depleted of basophils. Although a previous study demonstrated that ex vivo incubation of blood samples with prednisolone for 30 min did not significantly alter anti-IgE-mediated basophil activation [31], reduced basophil counts in blood following systemic corticosteroid treatment have been reported [66,67,68]. However, our study is the first to consider the impact of prolonged, systemic corticosteroid therapy on the circulating basophils from cancer patients.

Within our patient cohort, basophil activation, detected by CD63 cell surface up-regulation, was measurable in unfractionated whole blood samples within minutes following IgE-mediated activation (anti-FcεRI and anti-IgE), and/or for non-IgE-mediated activation (fMLP) (Figure 2). Basophils in 84.6% of samples responded to all stimuli with enhanced CD63 expression. We also identified patient blood samples in which basophils were activated either by IgE-dependent (5.8% of evaluable samples) or non-IgE-dependent (3.8% of evaluable samples) mechanisms, but not both. To our knowledge, this is the first report of basophils showing discrete capacities for activation by these widely investigated stimuli. Furthermore, basophils in 5.8% of evaluable samples were not activated by any of these stimuli (non-responders). “Non-responsiveness” of basophils has been attributed to dysregulation of signal transduction pathways downstream of FcεRI, especially in the kinases Syk and SHIP [69,70]. The incidence of basophil non-responders in our cancer patient cohort is similar to that described in other groups of patients whose basophils were subjected to IgE-mediated stimulation using the BAT [9,10], for example, in children evaluated for peanut allergy [13], tree and grass pollen allergies [8], and cow’s milk intolerance [71].

We considered a possible relationship between blood basophil function, serum tryptase and total IgE concentrations (Table S3). We found no correlation between the level of basophil activation and serum tryptase concentration in patients with serum tryptase concentrations within the normal range (2–14 ng/mL) or with the total serum IgE concentration (Figure S3). In a patient with elevated serum tryptase (33 ng/mL), the basophil population was comparable to that of the other patients (Figure 1), and CD63 up-regulation triggered by all stimuli was high (Figure 2 and Figure S3). Elevated basal serum tryptase can indicate mastocytosis or an increased risk of severe hypersensitivity reactions, such as to Hymenoptera venom [72,73,74,75], or tree nuts and peanuts [76], which could independently influence basophil function. However, basal serum tryptase is not chronically elevated in patients with sensitivity to nonsteroidal drugs or type I hypersensitivity to various other allergens [76,77,78]. The patient studied here did not have a diagnosis of mastocytosis, however, they also had a total serum IgE concentration above the reference range (466 kU/L, reference range 0–81 kU/L) (Figure S3), and a history of allergic diseases including asthma, which can be exacerbated by certain foods, and contact dermatitis. Although acute or chronic exposure of patients with allergies to relevant allergens might affect some of the functions of the blood basophils [79,80], this will require further investigation, since the atopic status of the other patients with ovarian cancer in the present study was not characterized. However, in our previous study of 42 ovarian cancer patients to evaluate potential hypersensitivity to a novel anti-cancer IgE therapeutic candidate, the one patient whose basophils were ex vivo activated by this therapy had a normal serum tryptase and total IgE concentrations (7 ng/mL and 39.2 kU/L, respectively). In the same study, basophils from the patient with elevated serum tryptase and high total serum IgE were not activated by the anti-tumor therapeutic IgE candidate [81]. Furthermore, in our recent publication of early data from the phase 1 clinical trial of this candidate (MOv18 IgE, ClinicalTrials.gov Identifier NCT02546921), we reported that the BAT is an effective monitoring companion to be used alongside other clinical safety parameters. When performed prior to IV infusion, BAT predicted hypersensitivity in the single patient who experienced anaphylaxis upon systemic exposure. This individual had no underlying allergic disease, and normal basal serum tryptase and total IgE concentrations [82]. It remains unclear, therefore, how serum tryptase, total IgE and atopic status in cancer patients may confound the capacity of their basophils to be activated by immune stimuli or therapeutic agents.

We investigated the possibility of targeted therapies [47,48,49,51,52,53,83], in addition to previous surgery and other chemotherapy, to diminish basophil numbers and activation. In our patients, despite prior treatment with surgery, chemotherapy and targeted therapies (anti-VEGF mAb bevacizumab, PARP inhibitors olaprarib and niraparib, or the anti-PD-L1 mAb avelumab), blood basophils were maintained and activation triggered by immune stimuli to degrees comparable to those measured in samples from other patients (Figure 3, Figure 4, and Figure S5). The degree of basophil activation was not dependent on the time, since the last targeted therapy treatment (Figure S5), although our observations are limited by patient numbers.

Furthermore, in concordance with case studies of hypersensitivity to chemotherapies [5,6,26,27,28,29], we detected blood basophil activation by paclitaxel in a patient who had previously experienced systemic reaction to therapeutic paclitaxel IV infusion. In addition to activation by IgE-mediated and non-IgE mediated stimuli, basophil CD63 expression was also elevated following incubation with paclitaxel, but not with carboplatin, a chemotherapy that the patient was known to tolerate. Basophil activation by paclitaxel may be triggered by a number of possible mechanisms [84]. Basophil activation could be (i) IgE-mediated, whereby IgE antibodies specific to paclitaxel or Cremaphor (CrEL; a polyethoxyated castor oil used in the formulation), are cross-linked upon subsequent exposure to these molecules [85]; (ii) non-IgE mediated, where paclitaxel or CrEL stimulates cells without the requirement for sensitization [86]; or (iii) by activation of complement [87,88]. The patient studied here experienced a serious systemic reaction to paclitaxel (including shortness of breath, back pain, and skin rash, followed by unresponsiveness, together symptoms in keeping with those reported for hypersensitivity elsewhere [84]) on their first infusion of neoadjuvant therapy for ovarian cancer. This suggested a mechanism not requiring sensitization. However, this individual had previously received treatment for another malignancy prior to diagnosis with ovarian cancer, so it may be possible that she was previously exposed to paclitaxel, CrEL or a biosimilar, resulting in sensitization. Nevertheless, we demonstrated hypersensitivity to paclitaxel in the BAT assay more than four years since the clinical observations of serious systemic reaction to paclitaxel infusion. This suggested that the hypersensitivity was maintained without repeated exposure and basophil reactivity was not altered by significant treatment with a range of therapies (including surgery, chemotherapies and niraparib) in the intervening period. Interestingly, at the highest concentrations of paclitaxel used for ex vivo stimulation, the degree of basophil activation was attenuated and the total basophil population in whole blood was markedly diminished. This may reflect deleterious activation of the basophils, mirroring the clinical manifestations of hypersensitivity to paclitaxel experienced by this patient (Figure 3 and Figure S4). Alternatively, it is possible that the paclitaxel preparation, containing CrEL, was toxic to the cells at these high concentrations. Others have previously described issues detecting or interpreting BAT results following incubation with high concentrations of chemotherapies [5]. Although, these include reactivity to therapies not prepared in CrEL and the study authors did not speculate why this was observed, nor describe the basophil population itself, making it difficult to compare directly with our observations. Future studies could make use of the BAT to elucidate mechanisms of hypersensitivity, including ex vivo stimulation with CrEL alone, or a CrEL-free formulation of paclitaxel such as Abraxane^®^. Regardless of the mechanism, our data support the utility of the BAT to monitor for hypersensitivity to therapeutic agents, even years after clinical adverse events, and may, therefore, be used to prevent potentially life-threatening reactions in sensitive individuals.

Our findings that basophils from patients with ovarian cancer retain their capacity for activation led us to consider whether basophils and their activation could be prognostic of patient outcomes. Higher percentage of basophils in whole blood samples from ovarian cancer patients was associated with improved overall survival (Figure 5A). Our data may be limited by the possible reduction in the proportion of basophils in whole blood samples by aggressive chemotherapy treatment and associated neutropenia. However, as shown in Figure 1E, we observed no significant change in the circulating basophil population relative to the time since prior chemotherapy treatment. In addition, the patients in our cohort had a broad spectrum of tumor histologies, and at the time of basophil analysis, some were treatment-naïve while others had previously received a range of anti-cancer treatments (Table S3). While these factors are likely to exert a greater influence on survival than a protective effect of basophils, our observations are similar to those of others: higher pre-operative basophil counts were associated with improved survival outcomes and measurements of less aggressive disease in colorectal cancer patients [2]; mice with higher basophil counts developed fewer and smaller lung metastases in a breast cancer model [89]. Furthermore, patients in our cohort with circulating basophils with a higher capacity for ex vivo activation detected by BAT also lived longer (Figure 5B).

We then considered whether basophils are found in ovarian tumors and whether these may also be prognostic of patient survival outcomes. Both protein and gene expression of markers indicative of basophils (CCR3, CD123, FcεRI) and basophil activation (CD63, CD203c, tryptase) were measured (Figure 6), indicating that basophils are found in ovarian tumors and that they may be in an activated state, which could potentially impact tumor-progression and patient outcomes. We therefore interrogated possible associations between gene expression for basophil markers in ovarian tumors and patient survival outcomes. This showed that high expression of activated basophil signatures was associated with improved progression-free and overall survival (Figure 7).

These observations, showing for the first time associations between basophils and ovarian cancer patient outcomes, are in keeping with reported activities of basophils in models of, or patients with melanoma [4], breast [90], and colorectal cancer [2,91]. However, the mechanisms through which basophils may be beneficial in cancer outcomes are not well-understood. Activated basophils release mediators, such as granzyme B, TNFα and histamine [90,92], which may act directly to regulate tumor growth. For instance, histamine could act directly on breast cancer cells, resulting in improved survival in a mouse model of breast cancer [93]. Basophils may also interact with other immune cells in networks leading to combined anti-tumoral effects. Basophils reportedly interact with and stimulate B cells through CD40L and release of IL-4, IL-6, IL-13, BAFF, and histamine, all of which may augment B cell proliferation, survival and antibody responses against cancer [94,95]. Basophil release of chemokines, such as CCL3 and CCL4, are also thought to play a role in attracting T cells into tumors, which in a melanoma mouse model, led to tumor rejection [4]. Contrastingly, basophils may exert pro-tumor activities, through release of pro-angiogenic and lymphangiogenic mediators, such as VEGF-A and VEGF-B, CXCL8, angiopoietin-1, hepatocyte growth factor, and tryptase [92,96,97], and may suppress anti-tumoral immune responses, such as through T-reg interactions [98,99]. These opposing roles of basophils are yet to be fully elucidated in the context of specific cancers. Future studies may include further investigation of basophil prognostic values in immunocompetent and basophil-deficient tumor-bearing animals, however, appropriate models that best represent human basophil immunity, localization and activation in cancers must be explored, and be supported by clinical observations.

In conclusion, we have demonstrated that circulating basophils in samples from a broad range of patients with ovarian cancer can be detected and activated ex vivo in response to a range of stimuli. The capacity for activation of cancer patient-derived basophils is consistent with those of widely studied human populations with allergies to food, venom and therapeutic drugs. Cancer patient-derived basophil activation ex vivo is also consistent with clinical observations of hypersensitivity to chemotherapy. This observation, in addition to our previous report that the basophil activation test predicted hypersensitivity to the first anti-tumor IgE therapeutic candidate MOv18 IgE, suggests that basophils may serve as predictive or monitoring tools for the development of hypersensitivity to therapeutic agents in oncology (AllergoOncology). Moreover, basophils and their activation may be associated with improved outcomes for ovarian cancer patients. Future studies can further explore the active state of these rare blood cells to better understand their responses to allergenic stimulation and cancer-associated immunomodulatory signals.

## Supplementary Materials

The following are available online at https://www.mdpi.com/2073-4409/9/7/1631/s1, Table S1: Antibodies used in immunohistochemistry (IHC) (The Protein Atlas online tool), Table S2: Gene expression probes and datasets used in survival analyses (KM Plotter online tool), Table S3: Ovarian cancer patient demographic and basophil characteristics, Figure S1: Basophil activation following blood collection, Figure S2: Basophils in whole blood and time lapse since last chemotherapy treatment, Figure S3: Serum tryptase and total IgE, Figure S4: Hypersensitivity to chemotherapy confirmed by ex vivo basophil stimulation, Figure S5: Circulating basophils and time lapse since last targeted therapy treatment, Figure S6: Tryptase and patient outcomes.
