# Supplementary material for: Basophils from Cancer Patients Respond to Immune Stimuli and Predict Clinical Outcome
Source: Cells. 2020 Jul 7;9(7):1631. doi: 10.3390/cells9071631 (PMC7408103; doi:10.3390/cells9071631)
Supplement: Supplementary file 1 [file cells-09-01631-s001.pdf]

Supplementary Material

Table S1 – Antibodies used in immunohistochemistry (IHC) (The Protein Atlas online tool).

Marker	Antibody
CD123	CAB018374
CCR3	CAB003795
FcεRI	HPA036051
CD63	CAB026356
CD203c	HPA043772
Tryptase	CAB016369

Table S2 – Gene expression probes and datasets used in survival analyses (KM Plotter online tool).

Marker	Gene Probe ID
CD123	206148_at
CCR3	208304_at
FcεRI	211734_s_at
CD63	200663_at
CD203c	232737_S_at
Tryptase	210084_x_at
**Datasets included in analyses**	
GSE14764	
GSE15622	
GSE18520	
GSE19829	
GSE23554	
GSE26193	
GSE26712	
GSE27651	
GSE30161	
GSE3149	
GSE51373	
GSE63885	
GSE65986	
GSE9891	
TCGA	

Table S3 – Ovarian cancer patient demographic and basophil characteristics.

	Characteristics and treatment history			Serum tryptase (ng/ml)	Total serum IgE (kU/L)	% Basophils	BAT Fold change in %CD63		
	Age	Tumor Histology	Previous treatment				anti-FcεRI	anti-IgE	fMLP
1	66	HG serous	Surgery and chemotherapy	4	34.8	0.45	9.9	5.6	7.1
2	52	HG serous	Surgery, chemotherapy and bevacizumab	6	61.3	0.62	18.7	11.6	15.6
3	82	Serous	Surgery and chemotherapy	6	6.2	0.52	50.1	51.2	7.4
4	66	HG serous	Surgery, chemotherapy and bevacizumab	9	8.7	0.39	59.0	55.0	5.5
5[a]	67	HG serous	Surgery, chemotherapy and prolonged oral corticosteroids	3	2.9	0.01	-	-	-
6[b]	58	HG serous	Surgery and chemotherapy	12	19.2	1.02	16.4	10.2	1.8
7	60	HG serous	Surgery and chemotherapy	6	308	0.30	8.0	5.5	5.7
8[c]	56	HG serous	Surgery and chemotherapy	5	37.3	0.54	1.5	0.6	2.1
9	70	HG serous	No treatment	7	39.2	0.36	33.2	60.8	35.7
10	48	HG serous	Surgery, no chemotherapy	4	69.1	0.73	49.7	36.3	15.9
11	74	HG serous	Surgery and chemotherapy	7	15.9	0.50	10.9	12.4	11.5
12	65	HG endometrioid	Surgery and chemotherapy	7	30.7	0.25	39.3	26.8	15.7
13[b]	55	HG serous	Surgery and chemotherapy	4	24.6	0.14	31.3	16.0	2.5
14[c]	80	HG serous	Surgery and chemotherapy	4	120	0.17	1.6	1.3	2.4
15	55	HG clear cell	Surgery, chemotherapy and bevacizumab	2	518	0.30	11.0	5.3	7.9
16[c]	68	Mixed histology	Surgery and chemotherapy	5	381	0.02	0.6	1.0	2.3
17	47	HG serous	Surgery, no chemotherapy	4	47.7	0.64	45.6	50.8	16.7
18	66	HG serous	Surgery, no chemotherapy	8	9.3	0.48	64.0	58.2	26.7
19[d]	72	HG serous	Surgery and chemotherapy	<1	220	0.32	0.8	0.8	8.0
20	67	HG serous	Surgery and chemotherapy	7	94.3	0.61	5.3	3.8	21.1
21	53	HG serous	Surgery, no chemotherapy	6	18.0	0.60	41.1	29.3	27.2
22	60	LG serous	Surgery, no chemotherapy	3	51.5	1.32	32.5	15.0	9.1
23	74	Mixed histology	Surgery and chemotherapy	5	2.5	0.25	63.6	56.9	21.4
24	43	HG serous	No treatment	4	3.2	0.45	73.4	58.2	24.9
25	71	HG serous	No treatment	7	<2.0	0.38	27.0	42.3	18.4
26	79	HG serous	No treatment	3	11.8	0.58	76.9	71.3	20.9
27	47	HG serous	Surgery, no chemotherapy	4	75.4	0.63	31.0	23.3	24.0
28	48	LG endometrioid	No treatment	7	156	0.80	30.9	20.9	14.5
29	79	HG serous	No treatment	7	310	0.76	74.9	59.4	4.4
30	53	HG serous	Surgery and chemotherapy	-	-	0.40	76.2	42.5	9.8
31	61	HG serous	No treatment	14	91	1.01	10.7	11.3	5.5
32	62	HG serous	No treatment	6	9.6	0.22	14.2	3.7	8.8
33	54	HG serous	No treatment	-	20.9	0.79	46.1	14.8	10.9
34	71	Carcinosarcoma	Surgery, no chemotherapy	4	-	1.64	5.1	11.9	6.6
35	25	Mucinous	Surgery, no chemotherapy	6	-	1.16	6.5	4.3	4.3
36	62	HG serous	Surgery, chemotherapy and bevacizumab	6	-	1.58	9.4	4.1	25.9
37[b]	63	HG serous	Surgery, chemotherapy and bevacizumab	4	-	0.60	18.8	7.7	2.7
38	67	HG serous	Surgery and chemotherapy	3	46.6	0.36	33.5	22.3	29.5
39	71	HG serous	Surgery and chemotherapy	5	-	0.09	78.6	45.8	25.0
40	76	HG serous	Surgery, chemotherapy and bevacizumab	7	-	0.60	73.6	72.6	10.8
41	47	HG endometrioid	Surgery and chemotherapy	6	-	0.65	76.5	54.1	40.2
42[e]	61	HG serous	Surgery, chemotherapy and bevacizumab	33	466	0.76	87.4	82.3	56.6
43[d]	58	HG serous	Surgery, chemotherapy, bevacizumab and olaparib	-	115	0.53	1.9	1.2	11.9
44	59	HG serous	Surgery, chemotherapy and avelumab	-	-	0.56	78.6	75.4	9.5
45	68	Serous	Surgery and chemotherapy	-	40.9	0.52	70.9	13.8	43.4
46	52	HG serous	Surgery, chemotherapy and bevacizumab	-	-	0.74	86.9	84.8	5.2
47	56	Clear cell	Surgery and chemotherapy	-	-	1.20	93.3	87.2	29.3
48	56	HG serous	Surgery, chemotherapy and avelumab	-	-	0.42	97.5	85.1	12.5
49	62	HG serous	Surgery, chemotherapy and bevacizumab	-	-	0.60	93.7	75.2	48.8
50	61	HG serous	Surgery and chemotherapy	-	-	2.30	84.7	90.4	52.2
51	66	HG serous	Surgery, chemotherapy and bevacizumab	-	-	0.83	41.2	24.8	10.6
52	72	HG serous	Surgery and chemotherapy	-	-	0.63	85.9	57.9	33.5
53	56	HG serous	Surgery and chemotherapy and niraparib	-	7.8	0.76	41.1	5.4	7.9

[a]Patient who had received a recent prolonged course of high-dose oral corticosteroids and had an extremely low basophil count meaning BAT was not possible,
[b]Patients that responded to only IgE-mediated activation,
[c]'Non-responder' patients,
[d]Patients that responded only to non-IgE-mediated activation,
[e]Patient with elevated serum tryptase (ULN = 14 ng/ml) and total serum IgE (ULN = 81 kU/L).

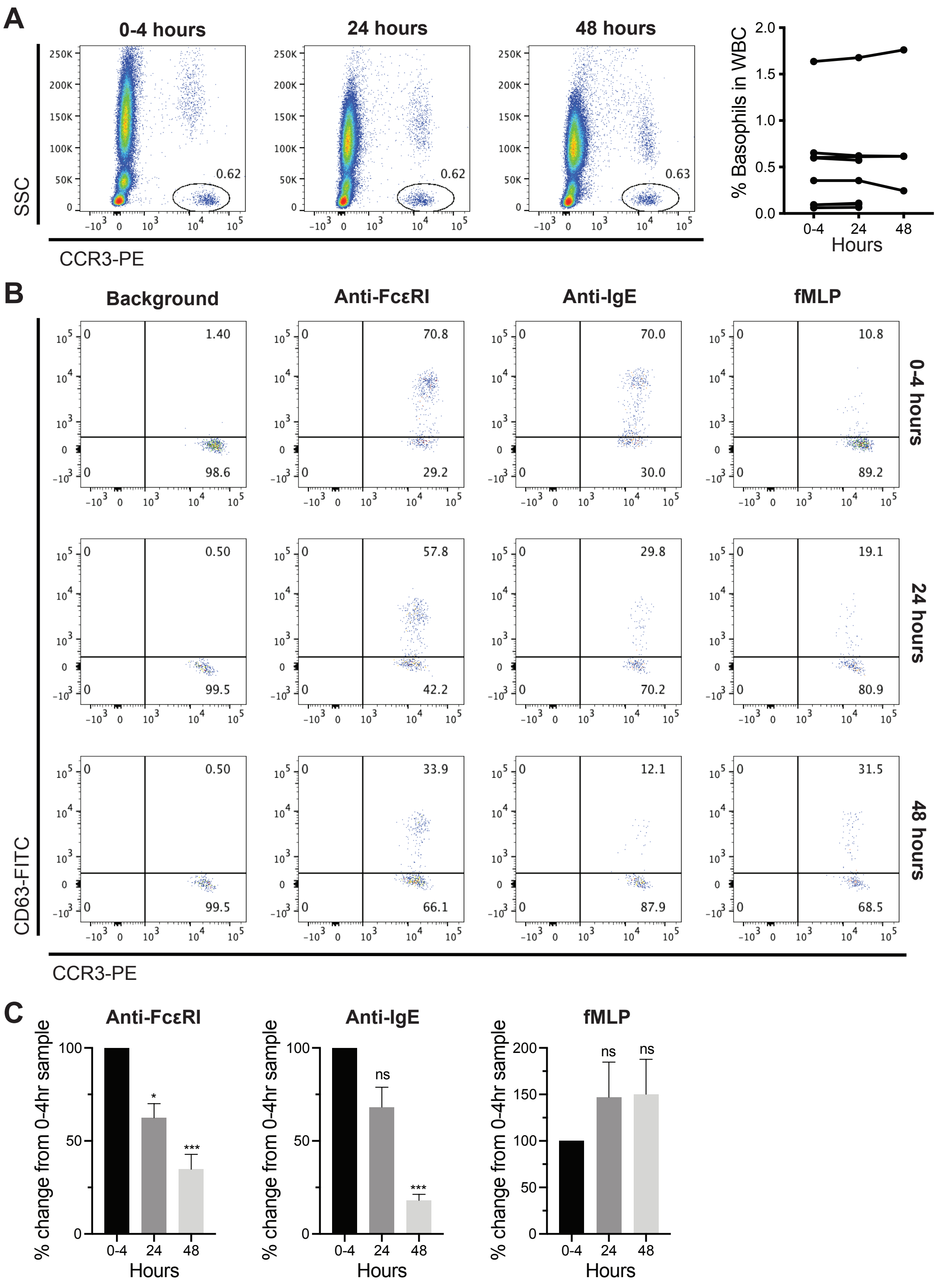

Figure S1 – Basophil activation following blood collection. Circulating CCR3^{high}SSC^{low} basophils identified by flow cytometry up to 4, 24 and 48 hours after blood collection (A) showed lower IgE-mediated activation compared to freshly-evaluated samples (B, C). Non-IgE-mediated activation was retained in samples stimulated 4, 24, and 48 hours after collection (B, C).

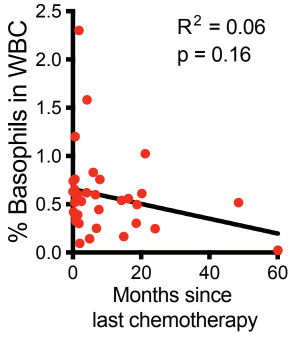

Figure S2 –Basophils in whole blood and time lapse since last chemotherapy treatment. No correlation was measured between the proportion of basophils in whole blood samples from ovarian cancer patients, and the time since they were last treated with chemotherapy.

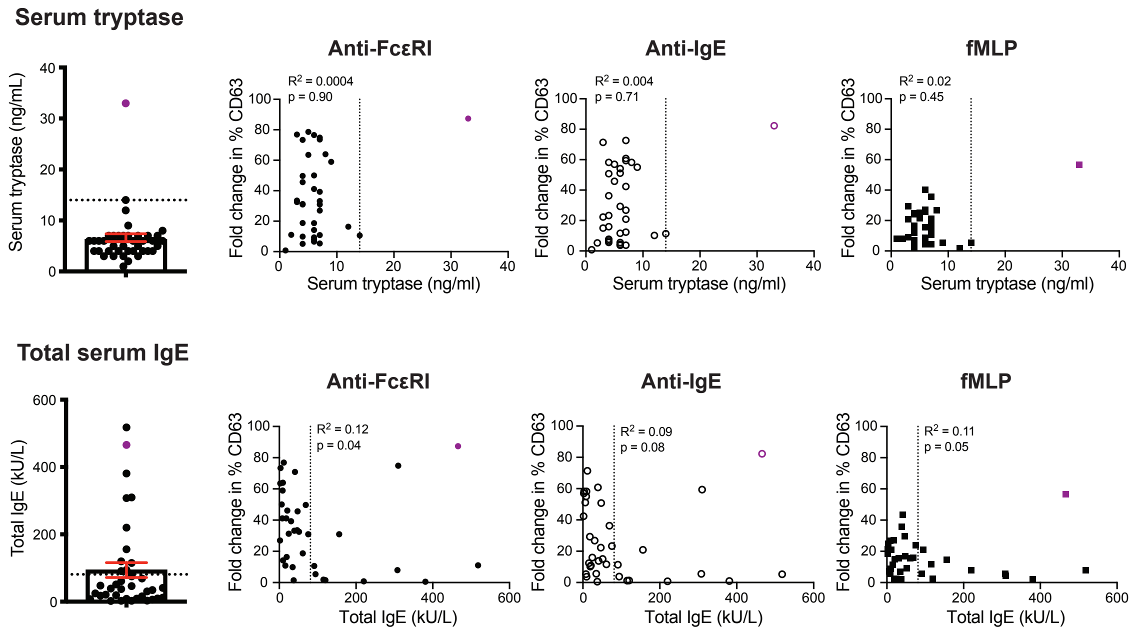

Figure S3 – Serum tryptase and total IgE. No correlation was measured between the level of *ex vivo* basophil activation and i. serum tryptase levels (in the normal reference range: 2-14 ng/ml), or ii. total IgE levels (reference range: 0-81 kU/L). Activation of basophils in blood from an ovarian cancer patient with elevated serum tryptase (33 ng/ml) and total IgE (466 kU/L) was high (purple symbols).

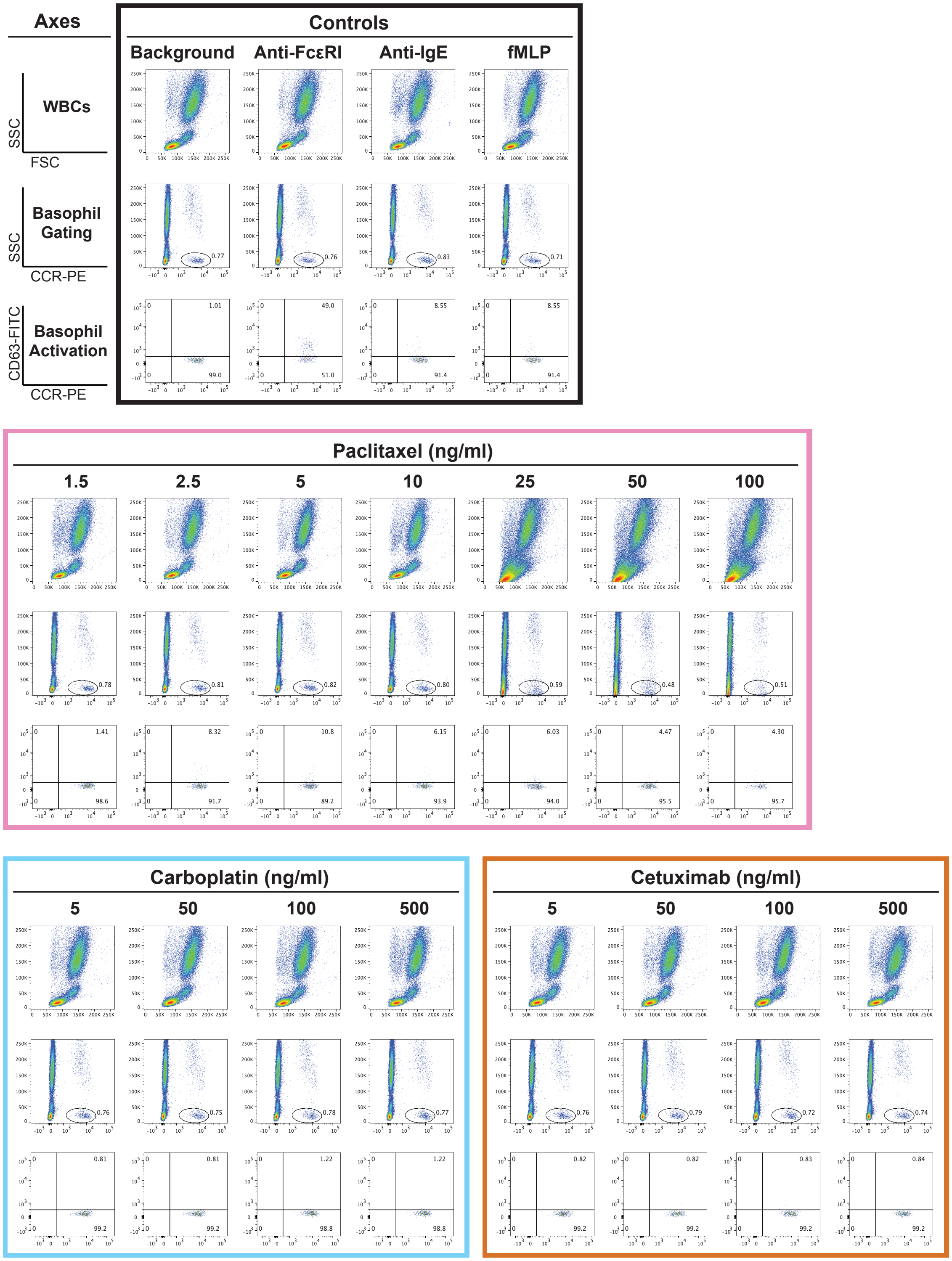

Figure S4 – Hypersensitivity to chemotherapy confirmed by *ex vivo* basophil stimulation. Flow cytometric dot plots of a blood sample from an ovarian cancer patient who had a prior systemic reaction following IV infusion of paclitaxel. Basophils were activated by *ex vivo* stimulation with anti-FcεRI, anti-IgE, fMLP, and paclitaxel (2.5–25 μg/ml). Following stimulation with concentrations of ≥25 ng/ml paclitaxel, marked changes to the WBC population were observed, clear identification of $CCR3^{high}SSC^{low}$ basophils was not possible, and measurement of cell-surface CD63 up-regulation was ablated, together suggesting a high basophil response. In contrast, circulating basophils from this patient were not activated by carboplatin (to which the patient is clinically tolerant), or cetuximab (an antibody with known hypersensitivity response in a subset of patients with cancer).

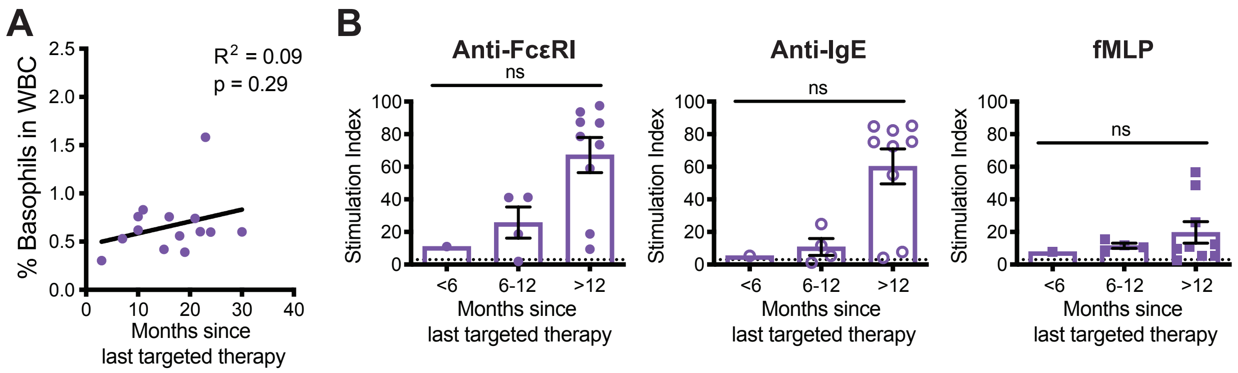

Figure S5 – Circulating basophils and time lapse since last targeted therapy treatment. No correlation was measured between the proportion of basophils in whole blood samples from ovarian cancer patients, and the time since they were last treated with targeted therapies (anti-VEGF mAb bevacizumab, PARP inhibitors olaprarib and niraparib, or the anti-PD-L1 mAb avelumab) (A). Similarly, the degree of basophil activation following *ex vivo* immune stimulation was independent of the time between the last treatment and BAT analysis (B).

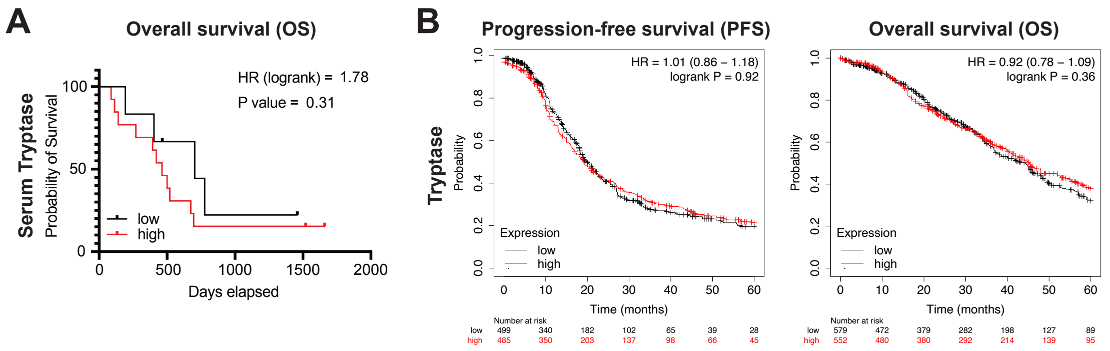

Figure S6 – Tryptase and patient outcomes. There is no association between ovarian patient survival outcomes and serum tryptase levels (A) or expression of tryptase in the tumor (B).
